# Evaluating the Maintenance of Lifestyle Changes in a Randomized Controlled Trial of the ‘Get Healthy, Stay Healthy’ Program

**DOI:** 10.2196/mhealth.5280

**Published:** 2016-05-10

**Authors:** Brianna S Fjeldsoe, Ana D Goode, Philayrath Phongsavan, Adrian Bauman, Genevieve Maher, Elisabeth Winkler, Elizabeth G Eakin

**Affiliations:** ^1^ School of Public Health Cancer Prevention Research Centre The University of Queensland Herston, Brisbane Australia; ^2^ School of Public Health The University of Sydney Sydney Australia

**Keywords:** maintenance, mHealth, physical activity, exercise, diet, nutrition, text message, behavior change

## Abstract

**Background:**

Extending contact with participants after initial, intensive intervention may support maintenance of weight loss and related behaviors.

**Objective:**

This community-wide trial evaluated a text message (short message service, SMS)-delivered, extended contact intervention (‘Get Healthy, Stay Healthy’ (GHSH)), which followed on from a population-level, behavioral telephone coaching program.

**Methods:**

This study employed a parallel, randomized controlled trial: GHSH compared with no continued contact (standard practice). Participants (n=228) were recruited after completing a 6-month lifestyle telephone coaching program: mean age = 53.4 (standard deviation (SD)=12.3) years; 66.7% (152/228) female; mean body mass index (BMI) upon entering GHSH=29.5 kg/m2 (SD = 6.0). Participants received tailored text messages over a 6-month period. The message frequency, timing, and content of the messages was based on participant preference, ascertained during two tailoring telephone calls. Primary outcomes of body weight, waist circumference, physical activity (walking, moderate, and vigorous sessions/week), and dietary behaviors (fruit and vegetable serves/day, cups of sweetened drinks per day, takeaway meals per week; fat, fiber and total indices from the Fat and Fiber Behavior Questionnaire) were assessed via self-report before (baseline) and after (6-months) extended contact (with moderate-vigorous physical activity (MVPA) also assessed via accelerometry).

**Results:**

Significant intervention effects, all favoring the intervention group, were observed at 6-months for change in weight (-1.35 kg, 95% confidence interval (CI): -2.24, -0.46, *P*=.003), weekly moderate physical activity sessions (0.56 sessions/week, 95% CI: 0.15, 0.96, *P*=.008) and accelerometer-assessed MVPA (24.16 minutes/week, 95% CI: 5.07, 43.25, *P*=.007). Waist circumference, other physical activity outcomes and dietary outcomes, did not differ significantly between groups.

**Conclusions:**

The GHSH extended care intervention led to significantly better anthropometric and physical activity outcomes than standard practice (no contact). This evidence is useful for scaling up the delivery of GHSH as standard practice following the population-level telephone coaching program.

## Introduction

Behavioral interventions can effectively promote weight loss [1,2]. Maintaining improvements in weight and related behaviors following the end of interventions is challenging [3-9]. Evidence from maintenance-focused interventions for physical activity, diet, and weight suggests the need for extended contact after initial intervention [10-12]. This contact can reinforce skills learned during the initial intervention, support ongoing problem solving, and provide continued accountability and motivation. Two meta-analyses of extended contact trials for weight loss concluded that they are viable and efficacious [12,13]. These meta-analyses (and other narrative reviews [14,15]) show that most extended contact interventions were delivered via face-to-face sessions, even though face-to-face session attendance decreases over time and as individuals regain weight [7,16]. There is some support for telephone-delivered extended contact interventions [16-20] and mixed evidence for Web-based extended contact interventions [12,21], with poor results being attributed to the lack of ongoing engagement with the website.

Text messages (short message service, SMS) may be a suitable, low-cost delivery modality for extended contact. It can: deliver tailored, repeated contacts; be actively “pushed” to participants; prompt behaviors in real time; and maintain two-way communication with an interventionist using minimal resources. Two recent pilot studies reported maintained weight loss in participants receiving a text message-delivered extended contact intervention [22,23]. This area of research has substantial promise for population health.

The Get Healthy Information and Coaching Service (GHS) is a population-wide telephone-delivered coaching program targeting weight loss, physical activity, and healthy eating in Australian adults [24]. Evaluations of the GHS have shown meaningful community-level weight loss and behavioral improvements at the end of the 6-month program [25] and, in a small subsample of participants, evidence of maintenance for weight loss and some behavioral outcomes, 6 months after completion [26].

The ‘Get Healthy, Stay Healthy’ (GHSH) randomized controlled trial compared a text message–delivered extended-contact intervention with standard practice (no contact) in GHS program completers. Primary outcomes for examining effectiveness of GHSH were: body anthropometry (weight, waist circumference), physical activity (self-reported walking, moderate and vigorous sessions/week, accelerometer-assessed moderate-vigorous physical activity minutes/week), and dietary behaviors (fruit and vegetable serves/day, cups of sweetened drinks/day, takeaway meals/week; fat, fiber, and total indices from the Fat and Fiber Behavior Questionnaire (FFBQ)). Secondary outcomes related to feasibility, acceptability, and costs of delivering GHSH.

## Methods

### Study Design

Data were collected at baseline (at GHS completion), 6 (end of GHSH extended contact), and 12 months (6 months following GHSH completion). Recruitment began in August 2012 and 6-month follow-up data collection were collected until March 2014. This paper reports on the baseline and 6-month data. Ethical clearance was received from the Human Research Ethics Committee at The University of Sydney (Protocol No.: 03-2011/13523). A detailed description of the trial methods is published elsewhere [27].

### Participant Recruitment

Eligibility criteria were: lives in New South Wales, Australia; no intention of re-enrolling in GHS coaching; not involved in other GHS evaluations; and owns a mobile telephone. All eligible clients completing the GHS within the recruitment timeframe were invited to participate during their final coaching call. Interested participants were mailed an information sheet and consent form and then contacted via telephone to establish their eligibility and willingness to participate. Verbal consent to participation was audio recorded and participants returned a signed consent form via reply paid post.

### Randomization

Participants were randomized 1:1 to GHSH intervention and control conditions, in two strata (GHS weight loss ≥ or < median of 3-kg loss), via a randomization website. This was done by a research assistant with no involvement in participant recruitment.

### The Get Healthy, Stay Healthy Intervention

The Get Healthy, Stay Healthy (GHSH) intervention was delivered via individually tailored text messages, with tailoring data collected during two telephone calls. Participants chose to focus on a weight loss or weight maintenance goal, and on physical activity or diet or both (with targets consistent with national guidelines) [28,29].

#### Initial Tailoring Call

This telephone call was scripted, and conducted by a trained health coach (not a GHS coach). Participants set a goal for weight maintenance or further loss within 3 months, and then two goals for behavior change. For each behavioral goal, they were asked to identify: rewards for reaching their goal; expected benefits; preparatory behaviors for goal attainment; barriers and solutions; and a person who could support them to reach their goals. Participants selected their desired number of text messages (from 3-13 per fortnight), timing of texts (eg, 6 AM), and type of texts (from the four types described below). This information was recorded during the call and was used to tailor GHSH texts.

#### Get Healthy, Stay Healthy Text Messages

Four types of texts targeted different behavior change strategies, each with different permitted frequencies (an example of each text type has been previously published [27]):

1. Prompts to self-monitor weight (once per fortnight).
2. Goal check text messages (from once per fortnight to once per week for each behavioral goal) that asked participants to reply “yes” or “no” to indicate their attainment of behavioral goals in the past week. Participants received a tailored goal check reply text message based on their yes/no response.
3. Real-time behavioral prompts (from none to four per fortnight for each behavioral goal) that remind participants of their goals, preparatory behaviors, and anticipated barriers and solutions.
4. Two goal reset text messages (one in week 6 and one in week 18) that prompt participants to consider their weight and behavioral goals and reset them appropriately. Participants were encouraged to tell their GHSH coach their new goals via reply text and these changes were reflected in subsequent texts.

Wording of the text messages (each ≤160 characters) was tailored to each individual’s name, gender, goals, identified barriers and strategies, preparatory behaviors to achieve their goals, expectations of behavioral change, and the first name of their identified support person. Texts were generated and sent by research staff, using a purpose-designed software package in which messages were preprogrammed in advance and scheduled to be sent at specific times. Replies to the goal check texts were stored and automatically triggered tailored responses whenever the participant replied with “yes,” “no,” or accepted variations of these. Whenever participants’ goal check reply contained additional words or an unrecognized variant of “yes” or “no” the program emailed research staff who manually decided which tailored reply to send. Unprompted reply text messages from participants did not receive a reply. At any stage participants, could change their text message preferences via text message or telephone call.

#### Twelve-Week Tailoring Call

At 12 weeks, participants received a second telephone call from their coach to update their tailoring information. This call was made between weeks 12 and 14, and if contact was not made during this period, the existing tailoring information was used for the final 12 weeks.

### Control Group Treatment

To minimize trial attrition, control participants were posted brief written feedback of results following each assessment. The control group received no other contact.

### Data Collection

The anthropometric and behavioral measurement tools used in this study were the same as those used in the GHS evaluation to enable comparison. More detailed data on MVPA and dietary behaviors were also collected at baseline and 6-months via: a computer-assisted telephone interview (CATI) conducted by a research assistant, a paper-based questionnaire, and a posted accelerometer. The research assistant was blinded to group allocation at baseline, but participants may have mentioned treatment during the 6-month CATI and so blinding was not guaranteed. Participants had previously provided demographic data and data on change in primary outcomes during the initial GHS.

### Primary Outcomes

#### Anthropometric Outcomes

During the CATI, participants reported their body weight in kilograms (while wearing light clothes and no shoes). They were encouraged to weigh themselves during the CATI if scales were present; otherwise, they were asked to report their most recent weighing. Participants were posted a measuring tape and instruction sheet on measuring waist circumference at baseline. The interviewer instructed participants to take the waist circumference measurement during the call. Body mass index (BMI) was calculated based on self-reported height at GHS baseline and self-reported weight at each assessment point. These self-report methods have been validated against objectively measured weight and waist circumference in a subsample of GHS users (n=38) [25]. This validation study showed strong correlations (Spearman rho >0.90) with the objective measures for both outcomes. There were 84% and 87% agreements in BMI and waist circumference classifications, respectively [25].

#### Physical Activity

During the CATI, participants completed a validated, 3-item assessment tool (3Q-PA) which asked about the number of weekly sessions spent: walking for ≥30 minutes; doing other moderate-intensity physical activity for ≥30 minutes (termed “moderate”); and, doing vigorous-intensity physical activity for ≥20 minutes [30].

Participants were also posted a belt-mounted dual-axis accelerometer (Actigraph model GT1M) initialized to collect data in 10-second epochs, a wear-time log, and a reply-paid envelope to return their materials. Participants were asked to wear the monitor on the hip for 7 consecutive days during all waking hours, and to remove the monitor only for sleep (if desired) and during times the monitor could be damaged (eg, during water-based activities). The wear log included monitor-fitting instructions and asked about any monitor removals, sleep time, and whether the monitor was worn or removed during sleep. Accelerometer data were downloaded in Actilife (v 6.6.2). Both 10- and 60-second epoch files were processed in SAS version 9.3. Nonwear time was excluded (ie, ≥60 minutes of 0 counts per minute (cpm), allowing for up to 2 minutes of 1 to 49 cpm [31]) and only days with ≥10 hours of wear were deemed valid. Data were plotted (as heatmaps) and compared against wear logs. Any sleep not already excluded as nonwear was excluded, along with any days that had registered as valid but the monitor was in the post. All minutes with ≥1952 cpm (vertical axis [32]) were classed as MVPA, then summed for each day and averaged across valid days.

#### Dietary Behaviors

During the CATI, participants reported the following: number of daily servings of fruit and of vegetables [33]; average daily consumption of sweetened drinks (cordials, fruit juices, sports drinks, soft drinks not including diet soft drinks); and takeaway meals per week [34]. Participants also completed the FFBQ [35], which asks about consumption of high-fat and high-fiber foods over the previous month concerning cooking, eating and food choice behaviors, and two items regarding fruit and vegetable intake. The average of the relevant items (1-5, with higher values indicating healthier habits) form the three FFBQ indices (20-item Total Index, 13-item Fat Index, and 7-item Fiber Index). These have good reliability, acceptable validity and good responsiveness to change [35].

### Secondary Outcomes

#### Feasibility Indicators

The text message software collected data on delivery (ie, number and type of text messages sent) and intervention engagement (ie, number of responses to goal check texts and achievement of weekly behavioral goals). The duration of the tailoring interviews and participant alterations to text preferences were tracked by research staff.

#### Acceptability Indicators

At 6 months, intervention participants rated satisfaction and usefulness in five categories (“not at all” to “extremely” satisfied/useful), of the GHSH intervention overall and specifically regarding support for achieving behavioral and weight loss goals. Intervention participants were invited to complete a telephone interview with research staff (approximately 10 minutes) involving open-ended questions regarding intervention usage, satisfaction, and potential program improvements. These interviews were audio-recorded, transcribed verbatim, and coded independently by two authors. Discrepancies between coding were discussed until consensus was reached and recruitment stopped once thematic saturation was reached.

#### Costs of Intervention Delivery

Personnel time was tracked for delivering all intervention-related tasks. Coach time was costed at AU$37.56 per hour and research staff time was costed at AU$31.56 per hour. Average duration per participant (minutes) was multiplied by the relevant personnel cost. Direct costs of sending the texts (at AU$0.15 each) were tracked, totaled and then divided by the total number of intervention participants to provide a per participant cost.

### Sample size

The sample size was chosen a priori to provide ≥90% power to detect the following expected differences between groups in primary outcomes: two sessions/week of self-reported MVPA; one serving of fruit per day and one serving of vegetables per day; 2-kg body weight; and 4-cm waist circumference as previously reported [27]. These were larger than the minimum differences of interest (MDI) in ascertaining that a group difference is not meaningful or change is less than a meaningful amount (maintained). MDIs were: 1-kg weight, 1-cm waist circumference, 30 minutes or 0.5 sessions/week physical activity, 0.5 servings/day fruit and vegetables, 0.5 takeaway meals/week, 0.25 cups/day sweetened drinks and 0.2 units on the FFBQ Indices. Power was adequate (≥80%) to detect these MDIs only for the dietary outcomes other than vegetables. The study was not powered a priori for questions concerning within-groups changes.

### Statistical Analysis

Analyses were performed in SPSS version 22 and STATA version 13. Significance was set at *P*<.05, two-tailed. Analyses were based on intention-to-treat principles, analyzing all participants (not subgroups) as randomized, but a small amount of missing data was excluded (completers’ analysis). Intervention effects (between-group differences, intervention minus control) were assessed using regression models that adjusted for baseline values of the outcome to control regression to the mean, the randomization strata (GHS weight loss ≥ or < median of 3-kg loss) and potential confounders that were associated with the outcome at *P*<.2 (Multimedia Appendix 1). The broader GHS evaluation reported mean changes; for comparability, we report paired *t*-tests for changes within groups during the GHSH trial (baseline to 6 months) and for prior changes during the GHS evaluation. Intervention effects and within-groups changes for MVPA (log-transformed) were assessed using generalized estimating equations (GEE) to account for repeated measures (daily), and adjusting for predictors of missing data ( *P*<.2) (Multimedia Appendix 1).

#### Sensitivity Analyses

Intervention effects were re-examined using multiple imputation by chained equations in STATA (m=20 imputations), adjusting for the same covariates as the completers models as well as predictors of missing data ( *P*<.2). Alternative models assessed whether conclusions were affected by model choice, as the main models sometimes failed to meet requisite assumptions. For count outcomes, intervention effects were retested using negative binomial models or zero-inflated negative binomial models when these fit the data better (Vuong test) [36]. The zero-inflated models were best suited to modelling the behaviors (physical activity, soft drink, and takeaway meal consumption) in which many participants did not engage.

#### Interpretation of Findings

It is complex to interpret results from extended contact interventions where “no change” in outcomes can be interpreted as a positive finding. Within-group changes (“worsened” or “improved”) and between-group differences (intervention “better” or “worse” than control) were only claimed when these were statistically significant. As nonsignificant findings can indicate either no change/difference or an insufficient sample size to yield a conclusive finding, we only described outcomes as “maintained” or as groups being “similar” if in addition to the finding being nonsignificant, the likely true effect size for the change/difference as indicated by the 95% confidence interval (CI) was also very small (<MDI).

## Results

### Participants

**Figure 1:**
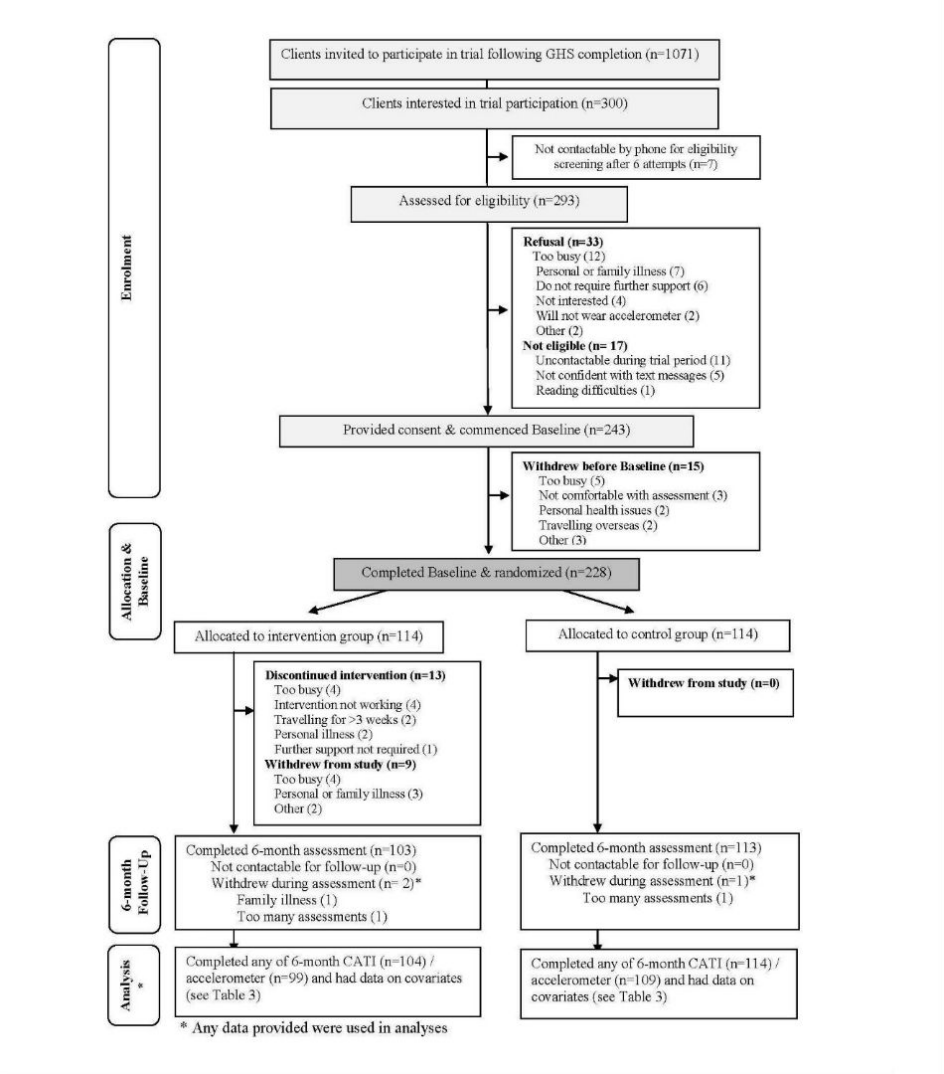
Participation in the Get Healthy, Stay Healthy (GHSH) trial.

There were 1071 clients invited to participate in this study, of whom 300 expressed verbal interest in participating in the trial, and 228 participants consented and were randomized (228/300, 76%; Figure 1). Table 1 shows the demographic characteristics of GHSH participants (intervention, n=114; control, n=114). Upon entering the GHSH trial, participants (152/228, 67% female) had a mean age of 53.4 (standard deviation (SD) = 12.3) years and BMI of 29.5 kg/m^2^(SD = 6.0), 34% (78/228) were overweight and 38% (87/228) were obese, 40% (91/228) did not meet physical activity guidelines [28] (ie, <150 minutes MVPA per week), and 80% (182/228) did not consume the recommended serves of fruit (ie, two) and/or vegetables (ie, five) per day [29].The GHSH sample was not representative of all participants completing the final GHS coaching call. Odds of being in the GHSH trial were significantly higher than their respective counterparts for those: aged <60 years; not working; without a post school qualification; self-reported diagnosis of hypertension; self-reported diagnosis of high cholesterol; doing no vigorous activity; and, who consume <1 takeaway meal per week (Multimedia Appendix 2).

The overall retention rate from baseline to 6 months was 95% (216/228) (103/114, 90% in intervention and 113/114, 99% in controls, *P*=.005). Most intervention participants (98/114, 86%) and controls (107/114, 94%) had full data on all outcomes at 6-month follow-up and on the baseline and prestudy covariates examined ( *P*=.077). Missing data was associated with: higher baseline BMI (mean ± SD for missing vs complete;

**Table 1:** Characteristics of ‘Get Healthy, Stay Healthy’ (GHSH) trial participants.

		Mean (SD) or n (%)	
		GHSH (n=114)^a^	Control (n=114)^a^
Health and demographics at baseline			
	Age (years)	55.5 (12.3)	51.2 (11.9)
	BMI (kg/m^2^)	29.3 (5.8)	29.6 (6.3)
	Weight (kg)	82.8 (19.4)	83.6 (18.9)
	Waist circumference (cm)	98.9 (15.4)	99.6 (14.9)
	Gender (% female)	74 (64.9%)	78 (68.4%)
	In paid employment (% yes)	69 (61.1%)	68 (59.6%)
	Education (% post-school qualification)	73 (64.0%)	77 (67.5%)
	Indigenous Australian (%)	1 (0.9%)	5 (4.4%)
	SEIFA^b^(% living in most advantaged 3 quintiles)^c^	86 (75.4%)	78 (68.4%)
	Region (% living in major cities)	71 (62.3%)	82 (71.9%)
Physical activity at baseline			
	Accelerometer PA^d^(minutes/week)	196.9 (144.4)	196.2 (143.6)
	Vigorous PA (sessions/week)	1.56 (1.86)	2.33 (2.53)
	Moderate PA (sessions/week)	1.11 (1.78)	1.60 (1.97)
	Walking PA (sessions/week)	3.99 (3.04)	3.30 (2.44)
Dietary behaviors at baseline			
	Vegetables (servings/day)	3.1 (1.4)	3.4 (1.8)
	Fruit (servings/day)	2.0 (0.9)	2.0 (1.0)
	Sweetened drinks (cups/day)	0.2 (0.5)	0.4 (0.9)
	Takeaway (meals/week)	0.5 (0.8)	0.5 (0.9)
	FFBQ Total Index (Score (1-5)	3.3 (0.4)	3.3 (0.4)
	FFBQ Fat Index (Score (1-5)	3.5 (0.5)	3.5 (0.5)
	FFBQ Fiber Index (Score (1-5)	2.9 (0.5)	2.9 (0.5)
Prior changes during GHS			
	Weight (kg)	−3.7 (4.0)^e^	−3.8 (4.3)^e^
	Waist circumference (cm)	−5.5 (5.0)^e^	−6.3 (5.7)^e^
	Vigorous PA (sessions/week)	0.5 (1.4)^e^	0.6 (1.7)^e^
	Moderate PA (sessions/week)	0.5 (2.1)^e^	0.9 (2.2)^e^
	Walking PA (sessions/week)	1.7 (2.7)^e^	1.1 (2.6)^e^
	Vegetables (servings/day)	1.2 (1.5)^e^	1.2 (1.4)^e^
	Fruit (servings/day)	0.6 (1.2)^e^	0.7 (1.1)^e^
	Sweetened drinks (cups/day)	−0.2 (0.6)^e^	−0.3 (1.2)^e^
	Takeaway (meals/week)	−0.5 (1.6)^e^	−0.5 (1.5)^e^

^a^Figures exclude missing data: one GHSH intervention participant (employment, English spoken at home, referral source, accelerometer PA) and one control participant (waist circumference, Indigenous status).

^b^Socioeconomic Indexes for Areas.

^c^Specifically the Index of Relative Socio-Economic Advantage and Disadvantage.

^d^Physical activity.

^e^Statistically significant change during GHS ( *P*<.05) based on *t*-test.

32.7 ± 7.3 vs 29.1 ± 5.8 kg/m^2^, *P*=.006); higher baseline weight (90.8 ± 19.5 vs 82.3 ± 18.9 kg, *P*=.044); higher rates of Type 2 Diabetes at baseline (8/23, 34.8% vs 22/205, 10.7%, *P*=.004); higher rates of smoking at baseline (5/23, 21.7% vs 7/205, 3.4%, *P*=.003); and lower vegetable intake at baseline (2.5 ± 1.1 vs 3.4 ± 1.6 servings per day, *P*=.012) (Multimedia Appendix 3). Participant’s reasons for discontinuing the intervention are shown in Figure 1, with the most common reasons being that they were too busy (4/13) or that the intervention was not working for them (4/13).

### Anthropometric and Behavior Change Results

#### Anthropometric Outcomes

Intervention participants showed significant reductions in both body weight (−0.89 kg, 95% CI: −1.53, −0.25) and waist circumference (−1.34 cm, 95% CI: −2.31, −0.36; Table 2). Intervention group improvements were significantly greater than controls for weight loss (−1.35 kg, 95% CI: −2.24, −0.46, *P*=.003), but not for waist circumference ( *P*=.115; Table 3).

#### Physical Activity

The intervention group maintained their accelerometer-assessed MVPA and self-reported vigorous activity, and changed significantly only in self-reported walking (−0.55, 95% CI: −0.99, −0.11 sessions/week; Table 2). The control group changed significantly in moderate activity and MVPA, which both declined (−0.68, 95% CI: −1.11, -0.26 sessions/week, and −16.10, 95% CI −28.60, −3.61 min/week; Table 2). The intervention group did significantly better than the control group in moderate activity (0.56, 95% CI: 0.15, 0.96 sessions/week) and in accelerometer-assessed MVPA (24.16, 95% CI: 5.07, 43.25 min/week; Table 3). Intervention effects for walking and vigorous activity were not significant (Table 3).

#### Dietary Behaviors

In the intervention group, small, but statistically significant improvements in dietary outcomes were observed for the FFBQ Fiber and Total Indices, while remaining dietary outcomes, except takeaway meals per week, were all maintained (Table 2). By contrast, in the control group, there were no statistically significant improvements, only statistically significant declines in vegetable intake and fruit intake (Table 2). Other dietary outcomes were maintained. No significant or meaningful intervention effect for dietary outcomes was observed.

#### Sensitivity Analyses

Results from analyses using multiple imputation mostly supported the interpretations from the main analyses (Multimedia Appendix 4), except that due to slightly narrower CIs in the multiple imputation analysis, the intervention group maintained takeaway meals/week (rather than being ‘inconclusive’) and the intervention effect for vegetable serves/day was ‘similar’ (rather than ‘inconclusive’). Conclusions using the alternative models mostly supported the main findings (Multimedia Appendix 5) and revealed significant differences in walking ( *P*=.018). The intervention group showed a greater tendency to walk than controls (odds ratio = 2.17, 95% CI: 0.45, 10.43, *P*=.334), but also to report fewer walking sessions when they did walk than controls (relative rate = 0.77, 95% CI: 0.64, 0.92, *P*=.005).

### Feasibility Results

The mean (±SD) call duration was 34 ±8 minutes for the initial tailoring call and 18 ±8 minutes for the 12-week tailoring call. The median number of text messages requested during the initial tailoring call and at the 12-week tailoring call were both five per fortnight (25^th^-75^th^percentiles =3-7 texts). During the initial tailoring call, 40% (46/111) of participants requested fortnightly goal checks for both behavioral goals; 35% (40/111) weekly for both goals; and, 22%(25/111) weekly for one goal and fortnightly for the other goal. Approximately one-half the participants (58/111, 52%) did not request any real-time behavioral prompts and 35% (39/111) requested between 2-4 prompts per fortnight. Almost all (89/95, 94%) intervention participants still enrolled at 12-weeks completed the 12-week tailoring interview. During the 12-week tailoring call, almost all (82/89, 92%) participants changed their preference for text message content, and 40% (36/89) changed their preferred text message schedule. Outside of the 12-week tailoring call, only 3% (3/90) of participants changed either of these preferences via text. The weight goal reset text received replies from 30% (33/111) of participants at week 6 and 26% (25/95) at week 18. At week 6, 40% (44/111) of participants reset at least one behavioral goal; 29% (28/95) did so in week 18. Intervention completers (n=90) replied to 84% of goal check texts sent in week 1 (152/180) and 69% (125/180) of goal check texts sent in the last week.

### Acceptability Results

Most (69/99, 70%) participants rated GHSH as “useful” or “extremely useful” in supporting their weight goal, and 75% (74/99) reported that they were “satisfied” or “extremely satisfied” with the intervention. Follow-up interviews with 62 intervention participants revealed that: experience with text messaging prior to GHSH impacted on participants’ experience during the intervention; the GHSH program often exceeded participants’ expectations; and participants generally perceived the GHS and GHSH intervention to be one program. Reasons for liking GHSH focused on: the reminders provided by the text messages to reinforce what participants wanted to do; maintaining accountability; and increased awareness of behaviors in real time. The few participants who did not like aspects of the GHSH nominated text messages being too repetitive or automated as key reasons.

### Costs of Intervention Delivery Results

Health coach staff spent, on average, 35 minutes per participant conducting and preparing for the initial tailoring interview and 30 minutes for the 12-week tailoring interview. Research staff spent on average 31 minutes per participant entering tailoring data into the software following the initial tailoring call and 15 minutes per participant after the 12-week tailoring call. Research staff had to manually trigger responses to 813 goal check replies not recognized by the software (813/2071, 39%) of responses received), which took approximately 1 minute per response. During the intervention, 8518 text messages were sent to participants (at AU$0.15 each) totaling AU$1278 (averaging AU$11.21 per participant). Overall, it cost approximately AU$80.00 per participant to deliver the GHSH extended contact intervention.

**Table 2:** Mean changes within the ‘Get Healthy, Stay Healthy’ (GHSH) intervention group (I; n=104) and the control group (C; n=114)

			MDI	Group	Mean Change (6 months – baseline)^a^		Within−group interpretation
					Mean (95% CI)	*P*	
Anthropometry							
	Weight (kg)		1 kg	I	− *0.89 (−1.53, −0.25)* ^b^	*.007*	Improved
				C	0.30 (−0.35, 0.95)	.357	Maintained
	Waist circumference^c^(cm)		1 cm	I	− *1.34 (−2.31, −0.36)*	*.008*	Improved
				C	−0.32 (−1.47, 0.82)	.578	Inconclusive
Physical activity (PA)							
	Vigorous PA (sessions/week)		0.5 session	I	0.20 (−0.10, 0.50)	.183	Maintained
				C	−0.40 (−0.86, 0.05)	.084	Inconclusive
	Moderate PA (sessions/week)		0.5 session	I	0.19 (−0.16, 0.54)	.277	Inconclusive
				C	− *0.68 (−1.11, −0.26)*	*.002*	Worsened
	Walking PA (sessions/week)		0.5 session	I	− *0.55 (−0.99, −0.11)*	*.015*	Worsened
				C	0.31 (−0.40, 1.01)	.392	Inconclusive
	Accelerometer PA^d^(minutes/week)		30 minutes	I	7.41 (−5.61, 20.44)	.265	Maintained
				C	− *16.10 (−28.60, −3.61)*	*.012*	Worsened
Dietary behaviors							
	Vegetables (servings/day)		0.5 serves	I	−0.17 (−0.46, 0.12)	.237	Maintained
				C	− *0.41 (−0.71, −0.12)*	*.006*	Worsened
	Fruit (servings/day)		0.5 serves	I	−0.06 (−0.22, 0.10)	.482	Maintained
				C	− *0.22 (−0.39,−0.05)*	*.011*	Worsened
	Sweetened drinks (cups/ day)		0.25 cups	I	−0.00 (−0.08, 0.08)	.982	Maintained
				C	−0.04 (−0.19, 0.11)	.602	Maintained
	Takeaway (meals/week)		0.25 meal	I	−0.13 (−0.28, 0.02)	.379	Inconclusive
				C	−0.06 (−0.19, 0.07)	.079	Maintained
	FFBQ Total Index Score (1–5)		0.2 units	I	*0.07 (0.02, 0.12)*	*.011*	Improved
				C	0.01 (−0.05, 0.07)	.781	Maintained
	FFBQ Fat Index Score (1–5)		0.2 units	I	0.06 (−0.01, 0.13)	.082	Maintained
				C	0.03 (−0.04, 0.11)	.368	Maintained
	FFBQ Fiber IndexScore (1–5)		0.2 units	I	*0.08 (0.01, 0.16)*	*.028*	Improved
				C	−0.03 (−0.12, 0.06)	.493	Maintained

^a^Mean changes estimated by paired *t*-test within completers, or by marginal means from GEE models for daily accelerometer MVPA (which was back-transformed from the log scale and multiplied by 7 yield minutes/week).

^b^Italic values indicate statistical significance at P˂.05.

^c^n=103 GHSH group; n=112 control group (item missing data for waist circumference).

^d^n=99 GHSH group; n=108 control group; some participants did not wear the accelerometer.

**Table 3:** Mean differences in changes between the ‘Get Healthy, Stay Healthy’ (GHSH) intervention (I; n=104) and control groups (C; n=114).

		Mean difference (GHSH – control)^a^		Between-group interpretation
		Mean (95% CI)	*P*	
Anthropometry				
	Weight (kg)	− *1.35 (−2.25, −0.46)* ^b^	*.003*	significantly better
	Waist circumference^c^(cm)	−1.18 (−2.65, 0.29)	.116	inconclusive
Physical activity (PA)				
	Vigorous PA (sessions/week)^d^	0.15 (−0.34, 0.63)	.547	inconclusive
	Moderate PA (sessions/week)^d^	*0.55 (0.14, 0.96)*	*.008*	significantly better
	Walking PA (sessions/week)	−0.69 (−1.46, 0.08)	.077	inconclusive
	Accelerometer PA (mins/week)^e^	*24.16 (5.07, 43.25)*	*.007*	significantly better
Dietary behaviors				
	Vegetables (servings/day)	0.15 (−0.21, 0.50)	.408	inconclusive
	Fruit (servings/day)^d^	0.16 (−0.05, 0.37)	.133	similar
	Sweetened drinks (cups/day)	−0.05 (−0.19, 0.10)	.537	similar
	Takeaways (meals/week)	0.01 (−0.15, 0.18)	.864	similar
	FFBQ Total Index Score (1–5)	0.05 (−0.03,0.13)	.195	similar
	FFBQ Fat Index Score (1–5)	0.02 (−0.07, 0.12)	.615	similar
	FFBQ Fiber Index Score (1–5)	0.08 (−0.03, 0.19)	.147	similar

^a^Mean difference (β) with 95% CI, and *P*value from linear regression models, adjusted for baseline values of the outcome and confounders (listed in Multimedia Appendix 1).

^b^Italic values indicate statistical significance at P˂.05.

^c^n=102 GHSH group; n=112 control group (item missing data for waist circumference).

^d^n=103 GHSH group; n=114 control group (item missing data for vigorous PA, moderate PA and Fruit).

^e^Estimated using marginal means from GEE models of log-transformed daily MVPA (repeated term for “day”), adjusting for confounders, and correcting for regression to the mean using the method [37] of including the term for assessment (pre/post) and the assessment x group interaction, but not the conditional term for group. Estimates were back-transformed to the original scale, then multiplied by 7 to yield minutes per week. n=112 GHSH group; n=114 control group (all participants with data at either time point examined).

## Discussion

### Principal Findings

Overall, the GHSH intervention was feasible to deliver and acceptable to participants. It led to significantly better outcomes compared with the control group in weight loss and some forms of physical activity, but not in dietary behaviors. For most dietary outcomes, meaningful intervention effects were also unlikely, based on the CIs. The study findings were inconclusive due to insufficient sample size for vegetable intake, walking, vigorous activity and waist circumference.

The GHSH weight loss of 0.89 kg (95% CI: −1.53, −0.25) during extended care was better than or consistent with comparable extended contact interventions. Spark and colleagues [23], in a single-group group trial of breast cancer survivors (n=29), found when receiving text message extended contact, women on average regained 1.3 kg (95% CI: −0.5, 3.1 kg) over 6 months. Donaldson and colleagues [22], in a small (n=34), nonparallel controlled trial, found intervention participants lost significantly more weight than controls (−1.6 vs 0.7 kg, 95% CIs not reported) over 12 weeks of extended contact that involved both text messages and face-to-face group sessions. The magnitude of change observed in this trial is also congruent with the intervention effects summarized in two recent meta-analyses of extended contact interventions [12,13] delivered via other modalities (ie, not text messaging).

The GHSH intervention was designed to follow on from a telephone coaching program, in which participants developed a rapport with a health coach. It is positive to note that a semiautomated text message program (with two brief telephone contacts) maintained the perception of accountability and that most participants felt personal engagement with their coach. It is encouraging that participants updated their text message preferences throughout the trial, given that continued engagement is a known facilitator of maintenance [12]. Particularly encouraging was that this highly tailored contact was achieved at a relatively low cost, which supports the scalability of GHSH.

### Strengths

Strengths of this trial include: the range of outcomes evaluated to inform service delivery; the high retention rate (216/228, 95%); its conduct in partnership with the service delivery agents and funders; and, the rigorous research design embedded within real-world delivery [38]. In line with public health principles and how the GHS is delivered, all participants were recruited and analyzed in this trial regardless of whether they complied with national guidelines at baseline, strengthening the findings further. These strengths enabled trial findings to inform the uptake of GHSH as part of standard delivery of the GHS program. Future evaluation of the GHSH impact once implemented into practice will advance our understanding of its uptake and the outcomes that can be achieved when scaled up for population-wide delivery.

### Limitations

While self-report of anthropometric outcomes was a limitation, the measures showed acceptable validity against objective measures in a GHS subsample [25]. The study was adequately powered for some but not all outcomes. The differences between trial participants and others completing GHS during the recruitment period may indicate the types of groups willing to receive a text message-delivered intervention, but may also reflect biases in research participation and eligibility criteria. This comparison of the trial sample to graduating GHS users is appropriate given this is the intended audience of the extended contact program; however, it is also useful to consider whether GHS users are broadly representative of Australian adults. Previous evaluations [39] have shown that GHS users were representative in relation to education, employment status, Aboriginal status, and fruit and vegetable consumption, but there was a disproportionately high representation of women, which may be expected given evidence of their higher likelihood to access health advice and attempt to lose weight compared with men. In the current trial, there was minimal missing data from the baseline and 6-month data collection points. Our sensitivity analysis using multiple imputation showed that missing data had very minimal impact on the interpretation of our findings.

### Conclusions

The GHSH extended contact intervention was feasible to deliver, acceptable to GHS clients and led to significantly better outcomes than standard practice in weight loss and physical activity. Supporting individuals to maintain positive lifestyle changes through cost-effective programs is paramount to the success of the GHS and more broadly to public health in Australia.

## Appendix

Multimedia Appendix 1 Confounders considered and adjusted for in main and sensitivity analyses.

Multimedia Appendix 2 Odds of being in the ‘Get Healthy, Stay Healthy’ (GHSH) trial (n=228) within those completing the Get Healthy Service (GHS) during the GHSH trial recruitment period (n=1071).

Multimedia Appendix 3 Characteristics of ‘Get Healthy, Stay Healthy’ (GHSH) trial participants at baseline with and without missing data

Multimedia Appendix 4 Mean changes within the ‘Get Healthy, Stay Healthy’ (GHSH) intervention group (I; n=114) and the control group (C; n=114) using multiple imputation for missing data.

Multimedia Appendix 5 CONSORT-EHEALTH Checklist.

Multimedia Appendix 6 CONSORT-EHEALTH Checklist.

## Abbreviations

BMI: body mass index
CATI: computer-assisted telephone interview
CI: confidence interval
CPM: counts per minute
FFBQ: Fat and Fiber Behavior Questionnaire
GEE: generalized estimation equations
GHS: Get Healthy Information and Coaching Service
GHSH: ‘Get Healthy, Stay Healthy’
MDI: minimum differences of interest
MVPA: moderate-vigorous physical activity
SEIFA: Socioeconomic Indexes for Areas
SD: standard deviation

## References

[1] Wu T, Gao X, Chen M, van Dam R M (2009). Long-term effectiveness of diet-plus-exercise interventions vs. diet-only interventions for weight loss: a meta-analysis. Obes Rev.

[2] Wadden TA, Butryn ML, Wilson C (2007). Lifestyle modification for the management of obesity. Gastroenterology.

[3] Wing RR, Tate DF, Gorin AA, Raynor HA, Fava JL (2006). A self-regulation program for maintenance of weight loss. N Engl J Med.

[4] Dansinger ML, Tatsioni A, Wong JB, Chung M, Balk EM (2007). Meta-analysis: the effect of dietary counseling for weight loss. Ann Intern Med.

[5] Wadden TA, Butryn ML, Byrne KJ (2004). Efficacy of lifestyle modification for long-term weight control. Obes Res.

[6] Curioni CC, Lourenço PM (2005). Long-term weight loss after diet and exercise: a systematic review. Int J Obes (Lond).

[7] Riebe D, Blissmer B, Greene G, Caldwell M, Ruggiero L, Stillwell KM, Nigg CR (2005). Long-term maintenance of exercise and healthy eating behaviors in overweight adults. Prev Med.

[8] Wing RR, Phelan S (2005). Long-term weight loss maintenance. Am J Clin Nutr.

[9] Dombrowski SU, Avenell A, Sniehott FF (2010). Behavioural interventions for obese adults with additional risk factors for morbidity: systematic review of effects on behaviour, weight and disease risk factors. Obes Facts.

[10] Riebe D, Blissmer B, Greene G, Caldwell M, Ruggiero L, Stillwell KM, Nigg CR (2005). Long-term maintenance of exercise and healthy eating behaviors in overweight adults. Prev Med.

[11] Fjeldsoe B, Neuhaus M, Winkler E, Eakin E (2011). Systematic review of maintenance of behavior change following physical activity and dietary interventions. Health Psychol.

[12] Patidar SM, Perri MG, Middleton K M Ross (2012). The impact of extended care on the long-term maintenance of weight loss: a systematic review and meta-analysis. Obes Rev.

[13] Dombrowski SU, Knittle K, Avenell A, Araújo-Soares V, Sniehotta FF (2014). Long term maintenance of weight loss with non-surgical interventions in obese adults: systematic review and meta-analyses of randomised controlled trials. BMJ.

[14] Simpson SA, Shaw C, McNamara R (2011). What is the most effective way to maintain weight loss in adults?. BMJ.

[15] Turk MW, Yang K, Hravnak M, Sereika SM, Ewing LJ, Burke LE (2009). Randomized clinical trials of weight loss maintenance: a review. J Cardiovasc Nurs.

[16] Hughes SL, Seymour RB, Campbell RT, Desai P, Huber G, Chang HJ (2010). Fit and Strong!: bolstering maintenance of physical activity among older adults with lower-extremity osteoarthritis. Am J Health Behav.

[17] Butler L, Furber S, Phongsavan P, Mark A, Bauman A (2009). Effects of a pedometer-based intervention on physical activity levels after cardiac rehabilitation: a randomized controlled trial. J Cardiopulm Rehabil Prev.

[18] Barnett LM, Van BE, Eakin EG, Beard J, Dietrich U, Newman B (2004). Program sustainability of a community-based intervention to prevent falls among older Australians. Health Promot Int.

[19] Martinson BC, Sherwood NE, Crain AL, Hayes MG, King AC, Pronk NP, O'Connor PJ (2010). Maintaining physical activity among older adults: 24-month outcomes of the Keep Active Minnesota randomized controlled trial. Prev Med.

[20] Perri MG, Limacher MC, Durning PE, Janicke DM, Lutes LD, Bobroff LB, Dale MS, Daniels MJ, Radcliff TA, Martin AD (2008). Extended-care programs for weight management in rural communities: the treatment of obesity in underserved rural settings (TOURS) randomized trial. Arch Intern Med.

[21] Neve M, Morgan PJ, Jones PR, Collins CE (2010). Effectiveness of web-based interventions in achieving weight loss and weight loss maintenance in overweight and obese adults: a systematic review with meta-analysis. Obes Rev.

[22] Donaldson EL, Fallows S, Morris M (2014). A text message based weight management intervention for overweight adults. J Hum Nutr Diet.

[23] Spark LC, Fjeldsoe BS, Eakin EG, Reeves MM (2015). Efficacy of a Text Message-Delivered Extended Contact Intervention on Maintenance of Weight Loss, Physical Activity, and Dietary Behavior Change. JMIR Mhealth Uhealth.

[24] O'Hara BJ, Bauman AE, Eakin EG, King L, Haas M, Allman-Farinelli M, Owen N, Cardona-Morell M, Farrell L, Milat AJ, Phongsavan P (2013). Evaluation framework for translational research: case study of Australia's get healthy information and coaching service(R). Health Promot Pract.

[25] O'Hara BJ, Phongsavan P, Venugopal K, Eakin EG, Eggins D, Caterson H, King L, Allman-Farinelli M, Haas M, Bauman AE (2012). Effectiveness of Australia's Get Healthy Information and Coaching Service®: translational research with population wide impact. Prev Med.

[26] O'Hara BJ, Phongsavan P, Eakin EG, Develin E, Smith J, Greenaway M, Bauman AE (2013). Effectiveness of Australia's Get Healthy Information and Coaching Service: maintenance of self-reported anthropometric and behavioural changes after program completion. BMC Public Health.

[27] Fjeldsoe B, Phongsavan P, Bauman A, Goode A, Maher G, Eakin E (2014). 'Get Healthy, Stay Healthy': protocol for evaluation of a lifestyle intervention delivered by text-message following the Get Healthy Information and Coaching Service®. BMC Public Health.

[28] Australian Government Department of Health and Ageing (2005). National Physical Activity Guidelines for Adults.

[29] Australian Government Department of Health and Ageing and National Health and Medical Research Council (2013). Eat for Health: Australian Dietary Guidelines, Summary.

[30] Smith BJ, Marshall AL, Huang N (2005). Screening for physical activity in family practice: evaluation of two brief assessment tools. Am J Prev Med.

[31] Gardiner PA, Clark BK, Matthews CE, Owen N, Healy GN, Winkler Elisabeth A H (2012). Identifying sedentary time using automated estimates of accelerometer wear time. Br J Sports Med.

[32] Freedson PS, Melanson E, Sirard J (1998). Calibration of the Computer Science and Applications, Inc. accelerometer. Med Sci Sports Exerc.

[33] Australian Bureau of Statistics and Australian Government Department of Health and Family Services (1995). National Nutrition Survey: User's Guide.

[34] New South Wales, Population Health Division (2009). New South Wales Adult Population Health Survey.

[35] Reeves MM, Winkler EA, Eakin EG (2014). Fat and fibre behaviour questionnaire: reliability, relative validity and responsiveness to change in Australian adults with type 2 diabetes and/or hypertension. Nutrition & Dietetics.

[36] Slymen DJ, Ayala GX, Arredondo EM, Elder JP (2006). A demonstration of modeling count data with an application to physical activity. Epidemiol Perspect Innov.

[37] Twisk J (2013). Applied Longitudinal Data Analysis for Epidemiology: A Practical Guide. Second Edition.

[38] Green LW (2008). Making research relevant: if it is an evidence-based practice, where's the practice-based evidence?. Fam Pract.

[39] O'Hara BJ, Phongsavan P, Venugopal K, Bauman AE (2011). Characteristics of participants in Australia's Get Healthy telephone-based lifestyle information and coaching service: reaching disadvantaged communities and those most at need. Health Educ Res.

