# Supplementary material for: Evaluating the Maintenance of Lifestyle Changes in a Randomized Controlled Trial of the ‘Get Healthy, Stay Healthy’ Program
Source: JMIR Mhealth Uhealth. 2016 May 10;4(2):e42. doi: 10.2196/mhealth.5280 (PMC4879328; doi:10.2196/mhealth.5280)
Supplement: Multimedia Appendix 1 [file mhealth_v4i2e42_app1.pdf]

Multimedia Appendix 1. Confounders considered and adjusted for in main and sensitivity analyses

Outcome	Adjusted in analyses [a]
Linear regression & GEE	
Weight (kg)	T1 weight (kg); randomisation strata, gender; education
Waist circumference (cm)	T1 waist circumference (cm); randomisation strata; referral source; hypertension; type 2 diabetes.
Vigorous PA (sessions/week)	T1 vigorous activity (sessions/week); randomisation strata; self-rated health; employment; hypertension; smoking; T1 BMI
Moderate PA (sessions/week)	T1 moderate activity (sessions/week); randomisation strata; self-rated health; gender, hypertension, T1 BMI
Walking (sessions/week)	T1 walking (sessions/week), randomisation strata; employment, baseline BMI
Accelerometer PA (minutes/day) [b]	T1 accelerometer PA; randomisation strata; gender; employment; type 2 diabetes; age; smoking
FFBQ Total Index (1–5)	T1 FFBQ Total Index; randomisation strata
FFBQ Fat Index (1–5)	T1 FFBQ Fat Index; randomisation strata; hypertension
FFBQ Fibre Index (1–5)	T1 FFBQ Fibre Index; randomisation strata, hypertension
Vegetables (servings/day)	T1 vegetables (servings/day); randomisation strata; gender, hypertension; type 2 diabetes; smoking; T1 BMI
Fruit (servings/day)	T1 fruit (servings/day); randomisation strata; self-rated health; gender, employment; age; T1 BMI
Sweetened drinks (cups/day)	T1 sweetened drinks (cups/day); randomisation strata; education
Takeaways (meals/week)	T1 Takeaways (meals/week); randomisation strata; self-rated health; gender; cholesterol; hypertension
Count models (Sensitivity Analysis)	
Vegetables (servings/day)	T1 vegetables (serves/day); randomisation strata; gender
Fruit (servings/day)	T1 fruit (serves/day); randomisation strata
Sweetened drinks (cups/day)	T1 sweetened drinks (cups/day); randomisation strata; gender; education
Takeaways (meals/week)	T1 takeaways (meals/week); randomisation strata; cholesterol
Vigorous PA (sessions/week)	T1 vigorous activity (session/week); randomisation strata; self-rated health; employment; referral source; smoking; T1 BMI
Moderate PA (sessions/week)	T1 moderate activity (sessions/week); randomisation strata; employment; cholesterol
Walking (sessions/week)	T1 walking (sessions/week); randomisation strata; self-rated health; hypertension; T1 BMI

Abbreviations: MVPA = moderate to vigorous physical activity; T1 = baseline; FFBQ = Fat and Fibre Behaviour Questionnaire; GEE = generalized estimating equations

[a] Models adjusted for baseline (T1) values of the outcome and randomisation strata (≥ median/< median GHS weight loss) (regardless of significance), and other potential confounders that were significant at $P<0.2$ selected from the following: Gender

(male/female); Education (< Year 12 or equivalent /Year 12 or equivalent /Post School Qualification); Employment (Full Time/Part-time or casual/Retired/Home Duties or other); Self-rated health “fair/poor” at GHS Baseline (yes/no); Referral by health professional into GHS (yes/no); GHS goal to lose weight (yes/no); Self-reported diagnosis of high cholesterol in GHS screening (yes/no); Self-reported diagnosis of hypertension in GHS screening (yes/no); Self-reported diagnosis of type 2 diabetes (yes/no) in GHS screening; Smoking status at end of GHS (never/ex-/current); Age at GHS Baseline (years); T1 total physical activity (sessions/week); and, BMI (kg/m^2) at T1. BMI is omitted from weight and waist circumference models (these adjust for weight and waist circumference) and T1 total physical activity is omitted from other physical activity models (that already adjust for walking, vigorous activity, moderate activity or MVPA).

[b] Accelerometer PA (all ActiGraph 60-second epochs ≥1952 counts) is estimated from GEE of log transformed daily MVPA, repeated measures by day and timepoint, adjusting for T1 MVPA indirectly by applying method outlined by Twisk [37]
