# Supplementary material for: Evaluating the Maintenance of Lifestyle Changes in a Randomized Controlled Trial of the ‘Get Healthy, Stay Healthy’ Program
Source: JMIR Mhealth Uhealth. 2016 May 10;4(2):e42. doi: 10.2196/mhealth.5280 (PMC4879328; doi:10.2196/mhealth.5280)
Supplement: Multimedia Appendix 2 [file mhealth_v4i2e42_app2.pdf]

**Multimedia Appendix 2.** Odds of being in the Get Healthy Stay Healthy (GHSH) trial (n=228) within those completing the Get Healthy Service (GHS) during the GHSH trial recruitment period (n=1071)

| | **In GHSH?** | | **Odds Ratio (95% CI)** | ***P*** |
|---|---|---|---|---|
| | **yes** | **no** | | |
| ***Health & Demographics*** | | | | |
| Age ≥60 years | 227 | 842 | **0.40 (0.29. 0.54)** | **<0.001** |
| Gender (female vs male) | 228 | 843 | 1.10 (0.81, 1.51) | 0.527 |
| In paid employment | 228 | 843 | **2.79 (2.07, 3.77)** | **<0.001** |
| Post-school qualification | 227 | 841 | **2.22 (1.64, 3.01)** | **<0.001** |
| Speaks English at home | 228 | 843 | 1.74 (0.77, 3.91) | 0.182 |
| Indigenous Australian | 227 | 842 | 1.24 (0.49, 3.17) | 0.649 |
| SEIFA (high vs. low) [a] | 225 | 842 | 0.80 (0.54, 1.15) | 0.217 |
| Reside in Major city | 225 | 842 | 0.94 (0.70, 1.26) | 0.685 |
| “Fair/poor” initial health | 227 | 842 | 0.87 (0.62, 1.22) | 0.408 |
| Type 2 diabetes | 228 | 843 | 0.90 (0.58, 1.38) | 0.614 |
| Hypertension | 228 | 843 | **0.52 (0.38, 0.72)** | **<0.001** |
| High cholesterol | 228 | 843 | **0.58 (0.42, 0.80)** | **0.001** |
| Health professional referred | 227 | 842 | 0.87 (0.53, 1.44) | 0.596 |
| Current smoker [b] | 228 | 842 | 1.41 (0.71, 2.28) | 0.326 |
| ***Anthropometry*** | | | | |
| Lost ≥5kg since baseline | 225 | 836 | 1.13 (0.84, 1.54) | 0.421 |
| Body Mass Index ≥25 kg/m$^2$ | 224 | 839 | 0.80 (0.57, 1.12) | 0.194 |
| At risk waist circumference [c] | 225 | 832 | 1.00 (0.69, 1.44) | 0.997 |
| ***Physical activity*** | | | | |
| ≥1 vigorous session/week | 216 | 774 | **2.14 (1.53, 3.00)** | **<0.001** |
| ≥1 Moderate session/week | 215 | 810 | 0.95 (0.70, 1.28) | 0.726 |
| ≥3 walking sessions/week | 215 | 810 | 0.88 (0.64, 1.21) | 0.438 |
| ***Dietary Behaviors*** | | | | |

| | | | | |
|---|---|---|---|---|
| ≥5 vegetables servings/day | 220 | 833 | 0.75 (0.54, 1.03) | 0.078 |
| ≥2 fruit servings/day | 220 | 835 | 0.88 (0.60, 1.28) | 0.505 |
| ≥1 cup/day sweetened drinks | 220 | 781 | 1.03 (0.63, 1.70) | 0.970 |
| ≥1 takeaway meals/week | 218 | 788 | **1.49 (1.08, 2.06)** | **0.016** |

Missing data excluded; all odds ratios are odds of completion for those with the characteristic versus without the characteristic unless specified otherwise; all data collected during the final GHS coaching call

[a] Socio-Economic Indexes for Areas (SEIFA), specifically the Index of Relative Socio-Economic Advantage and Disadvantage (IRSAD) – three most advantaged versus two least advantaged quintiles

[b] Most recently known status at end of GHS

[c] Waist circumference classified as “at risk” (>94 cm in men or >80cm in women) versus not (≤94 cm in men or ≤80cm in women)
