# Supplementary material for: Evaluating the Maintenance of Lifestyle Changes in a Randomized Controlled Trial of the ‘Get Healthy, Stay Healthy’ Program
Source: JMIR Mhealth Uhealth. 2016 May 10;4(2):e42. doi: 10.2196/mhealth.5280 (PMC4879328; doi:10.2196/mhealth.5280)
Supplement: Multimedia Appendix 3 [file mhealth_v4i2e42_app3.pdf]

Multimedia Appendix 3. Characteristics of 'Get Healthy, Stay Healthy' (GHSH) trial participants at baseline with and without missing data

	With data (n=205)		Missing data (n=23)		*P* [a]
	n	% or mean (SD)	n	% or mean (SD)	
Health & Demographics					
Age (years)	205	53.5 (12.2)	23	52.3 (13.4)	.650
Body Mass Index (kg/m^2)	**205**	**29.1 (5.8)**	**23**	**32.7 (7.3)**	**.006**
Weight (kg)	**205**	**82.3 (18.9)**	**23**	**90.8 (19.5)**	**.044**
Waist circumference (cm)	205	104.8 (15.5)	22	98.7 (15.0)	.071
Gender (male)	69	33.7 %	7	30.4 %	.820
(female)	136	66.3 %	16	69.6 %	
In paid employment (yes)	127	62.0 %	10	45.5 %	.169
(no)	78	38.0 %	12	54.5 %	
Education (post-school qual)	134	65.4 %	16	69.6 %	.818
(none)	71	34.6 %	7	30.4 %	
Speaks English at home (yes)	199	97.1 %	21	95.5 %	.515 [b]
(no)	6	2.9 %	1	4.5 %	
Indigenous Australian (yes)	5	2.5 %	1	4.3 %	.477 [b]
(no)	199	97.5 %	22	95.7 %	
SEIFA (most advantaged 3 quintiles)	145	70.7 %	19	82.6 %	.328
(most disadvantaged)	60	29.3 %	4	17.4 %	
Region (in major cities)	139	67.8 %	14	60.9 %	.492
(other area)	66	32.2 %	9	39.1 %	
Initial health (fair/poor)	157	76.6 %	16	69.6 %	.448
(good/excellent)	48	23.4 %	7	30.4 %	
Type 2 diabetes (yes)	**22**	**10.7 %**	**8**	**34.8 %**	**.004**
(no)	**183**	**89.3 %**	**15**	**65.2 %**	
Hypertension (yes)	54	26.3 %	7	30.4 %	.629
(no)	151	73.7 %	16	69.6 %	
High cholesterol (yes)	55	26.8 %	5	21.7 %	.803
(no)	150	73.2 %	18	78.3 %	
Current smoker (yes)	**7**	**3.4%**	**5**	**21.7 %**	**.003** [b]
(no)	**198**	**96.6 %**	**18**	**78.3 %**	
Physical activity (PA)					
Accelerometer PA (mins/week)	205	197.4 (136.6)	22	188.9 (201.9)	.794
Vigorous PA (sessions/week)	205	2.0 (2.2)	23	1.9 (2.4)	.939
Moderate PA (sessions/week)	205	1.4 (1.9)	23	1.2 (1.7)	.713
Walking PA (sessions/week)	205	3.6 (2.7)	23	4.4 (3.4)	.200
Dietary Behaviors					
Vegetables (servings/day)	**205**	**3.4 (1.6)**	**23**	**2.5 (1.1)**	**.012**
Fruit (servings/day)	205	2.0 (1.0)	23	2.0 (1.1)	.876
Sweetened drinks (cups/day)	205	0.3 (0.6)	23	0.5 (1.5)	.098
Takeaways (meals/week)	205	0.5 (0.8)	23	0.8 (1.3)	.095
FFBQ Total Index Score (1-5)	205	3.3 (0.4)	23	3.2 (0.3)	.412
FFBQ Fat Index Score (1-5)	205	3.5 (0.5)	23	3.4 (0.5)	.443
FFBQ Fibre Index Score (1-5)	205	2.9 (0.5)	23	2.9 (0.4)	.718

Abbreviations: SEIFA (Socio-Economic Indexes for Areas, specifically the Index of Relative Socio-Economic Advantage and Disadvantage)

[a] *P*-value for difference between those with missing vs non-missing data in terms of independent samples t-test (means) or chi-square test (percentages)

[b] invalid chi-square test; minimum expected count <2
