# Supplementary material for: Evaluating the Maintenance of Lifestyle Changes in a Randomized Controlled Trial of the ‘Get Healthy, Stay Healthy’ Program
Source: JMIR Mhealth Uhealth. 2016 May 10;4(2):e42. doi: 10.2196/mhealth.5280 (PMC4879328; doi:10.2196/mhealth.5280)
Supplement: Multimedia Appendix 4 [file mhealth_v4i2e42_app4.pdf]

Multimedia Appendix 4: Mean changes within the 'Get Healthy, Stay Healthy' (GHSH) intervention group (I; n=114) and the control group (C; n=114) using multiple imputation for missing data

	MDI	Group	Change (6 months – Baseline) [a] Mean (95% CI)	*P*	Within-group interpretation
**Anthropometry**					
Weight *(kg)*	1kg	I	**-0.99 (-1.63, -0.35)**	**.003**	improved
		C	0.30 (-0.34, 0.94)	.353	maintained
Waist circumference *(cm)*	1cm	I	**-1.40 (-2.41, -0.40)**	**.006**	improved
		C	-0.32 (-1.44, 0.80)	.579	inconclusive
**Physical activity (PA)**					
Vigorous PA *(sessions/week)*	0.5 session	I	0.14 (-0.18, 0.45)	.400	maintained
		C	-0.40 (-0.85, 0.05)	.080	inconclusive
Moderate PA *(sessions/week)*	0.5 session	I	0.19 (-0.14, 0.53)	.262	inconclusive
		C	**-0.68 (-1.10, -0.27)**	**.001**	worsened
Walking PA *(sessions/week)*	0.5 session	I	**-0.70 (-1.16, -0.23)**	**.003**	worsened
		C	0.31 (-0.40, 1.00)	.388	inconclusive
Accelerometer MVPA[b] *(mins/week)*	30 mins	I	7.62 (-5.35, 20.58)	.250	maintained
		C	**-15.64 (-27.68, -3.59)**	**.011**	worsened
**Dietary behaviors**					
Vegetables *(servings/day)*	0.5 serves	I	-0.17 (-0.45, 0.12)	.249	maintained
		C	**-0.41 (-0.70, -0.12)**	**.005**	worsened
Fruit *(servings/day)*	0.5 serves	I	-0.08 (-0.24, 0.09)	.359	maintained
		C	**-0.20 (-0.38,-0.05)**	**.009**	worsened
Sweetened drinks *(cups/ day)*	0.25 cups	I	-0.00 (-0.08, 0.09)	.918	maintained
		C	-0.04 (-0.19, 0.11)	.602	maintained
Takeaway *(meals/week)*	0.25 meal	I	-0.08 (-0.21, 0.05)	.224	maintained
		C	-0.13 (-0.28, 0.01)	.075	maintained
FFBQ Total Index Score *(1–5)*	0.2 units	I	**0.07 (0.01, 0.12)**	**.013**	improved
		C	0.01 (-0.05, 0.07)	.780	maintained
FFBQ Fat Index Score *(1–5)*	0.2 units	I	0.06 (-0.01, 0.13)	.081	maintained
		C	0.03 (-0.04, 0.11)	.364	maintained
FFBQ Fibre Index Score *(1–5)*	0.2 units	I	**0.09 (0.01, 0.16)**	**.028**	improved
		C	-0.03 (-0.12, 0.06)	.489	maintained

Abbreviations: MDI = minimum difference of interest; CI = confidence interval FFBQ = Fat and Fibre Behaviour Questionnaire MVPA=moderate to vigorous physical activity

[a] Mean changes within groups (?) estimated using mixed models, missing data multiply imputed by chained equations m=20 imputations.

[b] Mean changes (?) estimated from GEE models for daily accelerometer MVPA (which was back-transformed from the log scale and multiplied by 7 to yield minutes/ week) adjusted for confounders and predictors of dropout in n=112 intervention and n=114 control.

Multimedia Appendix 4b: Mean differences in changes between the 'Get Healthy, Stay Healthy' (GHSH) intervention (I; n=114) and control groups (C; n=114) using multiple imputation for missing data

	**GHSH - Control** [a]		**Between-group interpretation**
	**Mean Difference (95% CI)**	***P***	
**Anthropometry**			
Weight *(kg)*	**-1.41 (-2.30, -0.50)**	**.002**	significantly better
Waist circumference *(cm)*	-1.16 (-2.64, 0.31)	.122	inconclusive
**Physical activity (PA)**			
Vigorous PA *(sessions/week)*	0.12 (-0.37, 0.61)	.639	inconclusive
Moderate PA *(sessions/week)* [c]	**0.50 (0.10, 0.90)**	**.015**	significantly better
Walking PA *(sessions/week)*	-0.74 (-1.50, 0.02)	.056	inconclusive
Accelerometer MVPA *(mins/week)* [b]	**24.39 (5.30, 43.48)**	**.012**	significantly better
**Dietary behaviors**			
Vegetables *(servings/day)*	0.13 (-0.22, 0.49)	.459	similar
Fruit *(servings/day)* [c]	0.16 (-0.05, 0.36)	.144	similar
Sweetened drinks *(cups/day)*	-0.05 (-0.19, 0.10)	.537	similar
Takeaways *(meals/week)*	0.01 (-0.15, 0.18)	.861	similar
FFBQ Total Index *Score (1–5)*	0.05 (-0.03,0.13)	.196	similar
FFBQ Fat Index *Score (1–5)*	0.02 (-0.07, 0.12)	.608	similar
FFBQ Fibre Index *Score (1–5)*	0.08 (-0.03, 0.19)	.148	similar

Abbreviations: CI = confidence interval FFBQ = Fat and Fibre Behaviour Questionnaire MVPA=moderate to vigorous physical activity

[a] Mean difference (?) with 95% confidence interval (CI), and *P*-value from linear regression models, adjusted for baseline values of the outcome and confounders (listed in Multimedia Appendix 1). Missing data imputed by multiple imputation (chained equations) m=20 imputations.

[b] Estimated using marginal means from GEE models of log-transformed daily MVPA (repeated term for "day"), adjusting for confounders, and correcting for regression to the mean using the method [39] of including the term for assessment (pre/post) and the assessment x group interaction, but not the conditional term for group. Estimates were back-

transformed to the original scale, then multiplied by 7 to yield minutes per week. n=112 GHSH group; n=114 control group. GEE models did not use multiple imputation.
