# Supplementary material for: Evaluating the Maintenance of Lifestyle Changes in a Randomized Controlled Trial of the ‘Get Healthy, Stay Healthy’ Program
Source: JMIR Mhealth Uhealth. 2016 May 10;4(2):e42. doi: 10.2196/mhealth.5280 (PMC4879328; doi:10.2196/mhealth.5280)
Supplement: Multimedia Appendix 5 [file mhealth_v4i2e42_app5.pdf]

Multimedia Appendix 5. Differences between intervention (GHSH) and control group in count outcomes for dietary behaviors and physical activity at 6-month follow-up, adjusted for baseline values and confounders [a]

	n	Marginal mean (95% CI)		Comparison [b]	Difference (GHSH vs Control)		
	**GHSH/control**	**GHSH**	**Control**		**RR or OR (95% CI)**	***P***	***Overall P[c]***
Physical activity (PA)							
Vigorous PA *(sessions/week)*	103/114	1.78 (1.38, 2.17)	1.59 (1.19, 1.99)	Yes/No	1.31 (0.61, 2.83)	.492	*.761*
				Amount	1.02 (0.80, 1.29)	.887	
Moderate PA *(sessions/week)*	103/114	1.41 (1.09, 1.73)	0.84 (0.60, 1.08)	Yes/No	**2.90 (7.06, 1.19)**	**.019**	***.015***
				Amount	1.10 (0.76, 1.58)	.606	
Walking *(sessions/week)*	104/114	2.90 (2.50, 3.30)	3.60 (3.10, 4.09)	Yes/No	2.17 (0.45, 10.43)	.334	***.018***
				Amount	**0.77 (0.64, 0.92)**	**.005**	
Dietary behaviors							
Vegetables *(servings/day)*	104/114	2.99 (2.65, 3.32)	2.80 (2.49, 3.11)	Overall	1.07 (0.91, 1.24)	.464	-
Fruit *(servings/day)*	104/114	1.85 (1.59, 2.11)	1.70 (1.46, 1.94)	Overall	1.09 (0.90, 1.32)	.392	-
Sweetened drink *(cups/day)*	104/114	0.27 (0.12, 0.42)	0.31 (0.14, 0.48)	Yes/No	0.50 (0.02, 16.36)	.588	*.719*
				Amount	0.85 (0.49, 1.51)	.694	
Takeaway *(meals/week)*	104/114	0.43 (0.21. 0.65	0.43 (0.21,0.64)	Yes/No	0.67 (0.14, 3.22)	.614	*.819*
				Amount	1.16	.569	

	(0.69, 1.96)		

[a] Models adjust for baseline values of the outcome and potential confounders significant at *P*<0.2 (Listed in Multimedia Appendix 1). From the negative binomal regression models, we report relative rates (GHSH mean/ control mean), while from the zero-inflated negative binomal models we report separately on relative rate (GHSH mean/ control mean) when the outcome is present (e.g., number of sessions of vigorous activity, when the participant does vigorous activity) as well as the odds (GHSH/control that the outcome is present (e.g., that the participant does vigorous activity)).

[b] Comparisons shown as overall relative rates (RR) from negative binomial models; otherwise separately as odds ratio (OR) for the presence (yes/no) of the outcome and relative rate (RR) for the amount (if the outcome is present), from zero-inflated negative binomial models.

[c] **Overall test of significance that groups differ in either the odds of the outcome or the amount (if the outcome is present).**
